# Clinical characteristics and risk factors of patients with severe COVID-19 in Jiangsu province, China: a retrospective multicentre cohort study

**DOI:** 10.1186/s12879-020-05314-x

**Published:** 2020-08-06

**Authors:** Songqiao Liu, Huanyuan Luo, Yuancheng Wang, Luis E. Cuevas, Duolao Wang, Shenghong Ju, Yi Yang

**Affiliations:** 1 Department of Critical Care Medicine, Jiangsu Provincial Key Laboratory of Critical Care Medicine, Zhongda Hospital, School of Medicine, Southeast University, Nanjing, 210009 China; 2 Department of Clinical Sciences, Liverpool School of Tropical Medicine, L3 5QA, Liverpool, UK; 3 Department of Radiology, Zhongda Hospital, School of Medicine, Southeast University, Nanjing, 210009 China

**Keywords:** COVID-19, SARS-CoV-2, Pneumonia, Severity, Severe, Critically ill

## Abstract

**Background:**

Coronavirus Disease-2019 (COVID-19) pandemic has become a major health event that endangers people health throughout China and the world. Understanding the factors associated with COVID-19 disease severity could support the early identification of patients with high risk for disease progression, inform prevention and control activities, and potentially reduce mortality. This study aims to describe the characteristics of patients with COVID-19 and factors associated with severe or critically ill presentation in Jiangsu province, China.

**Methods:**

Multicentre retrospective cohort study of all individuals with confirmed Severe Acute Respiratory Syndrome Coronavirus-2 (SARS-CoV-2) infections diagnosed at 24 COVID-19-designated hospitals in Jiangsu province between the 10th January and 15th March 2020. Demographic, clinical, laboratory, and radiological data were collected at hospital admission and data on disease severity were collected during follow-up. Patients were categorised as asymptomatic/mild/moderate, and severe/critically ill according to the worst level of COVID-19 recorded during hospitalisation.

**Results:**

A total of 625 patients, 64 (10.2%) were severe/critically ill and 561 (89.8%) were asymptomatic/mild/moderate. All patients were discharged and no patients died. Patients with severe/critically ill COVID-19 were more likely to be older, to be single onset (i.e. not belong to a cluster of cases in a family/community, etc.), to have a medical history of hypertension and diabetes; had higher temperature, faster respiratory rates, lower peripheral capillary oxygen saturation (SpO_2_), and higher computer tomography (CT) image quadrant scores and pulmonary opacity percentage; had increased C-reactive protein, fibrinogen, and D-dimer on admission; and had lower white blood cells, lymphocyte, and platelet counts and albumin on admission than asymptomatic/mild/moderate cases. Multivariable regression showed that odds of being a severe/critically ill case were associated with age (year) (OR 1.06, 95%CI 1.03–1.09), lymphocyte count (10^9^/L) (OR 0.25, 95%CI 0.08–0.74), and pulmonary opacity in CT (per 5%) on admission (OR 1.31, 95%CI 1.15–1.51).

**Conclusions:**

Severe or critically ill patients with COVID-19 is about one-tenths of patients in Jiangsu. Age, lymphocyte count, and pulmonary opacity in CT on admission were associated with risk of severe or critically ill COVID-19.

## Highlights

- About one-tenths of patients with COVID-19 in Jiangsu had severe or critically ill presentation
- All patients were discharged and no patients died by the end of study
- Risk of severe or critically ill COVID-19 patients increased with age
- Low lymphocyte count and high pulmonary opacity score in CT on admission were associated with high risk of severe or critically ill COVID-19

## Background

Coronavirus Disease 2019 (COVID-19), caused by the etiological agent Severe Acute Respiratory Syndrome Coronavirus-2 (SARS-CoV-2), was first reported from Wuhan, Hubei province, China, in December 2019. The World Health Organization (WHO) declared a pandemic the 11th March 2020 [1]. The COVID-19 pandemic have spread quickly from a focal outbreak to over 7410, 000 cases with more than 400, 000 deaths affecting more than 140 countries by the 12th June 2020 [2].

China had reported over 80, 000 confirmed cases by the 13th March 2020 [2]. Although the original epicentre was located in Wuhan, other provinces became affected in the following weeks. In a case series of the first 44,672 confirmed cases, 1023 patients had died, with a crude case fatality rate (CFR) of 2.3%, and mortality was higher among critically ill patients, who had a CFR of 49% [3]. In Hubei, the proportion of health workers with severe or critical ill COVID-19 was higher than in other provinces (10.4 and 7.0%, respectively) [3]. Compared to China, the crude CFR in South Korea is lower among both males (1.1%) and females (0.4%) [4], and the crude CFR in Australia (1.4%) [5] and in countries of the European Union (EU) and European Economic Area (EEA) was also lower (1.5%) [6], while the crude CFR in Italy was much higher (7.2%) [7].

COVID-19 initial symptoms are not specific, presenting with fever, and cough, which can then resolve spontaneously or progress to shortness of breath, dyspnoea, and pneumonia, leading to acute respiratory distress syndrome (ARDS), renal failure, coagulation dysfunction, multiple organ failure and death [8–13]. Demographic features (eg age and gender); epidemiological features (eg smoking and comorbidities); laboratory parameters (eg C-reactive protein, albumin, cytokine, lactate dehydrogenase, D-dimer, platelet, lymphocyte, and neutrophil); clinical manifestations (eg cough, expectoration, chest pain, and dyspnea); computer tomography (CT) image test results; and others, have been reported to be associated with the severity of COVID-19 in some small studies in China [14–20], and other countires [13, 21, 22].

Understanding the factors associated with COVID-19 disease severity could support the early identification of patients with high risk for severe/critically ill COVID-19 presentation and inform prevention and control activities and reduce mortality. Hubei was the COVID-19 epicentre of China at the time of data collection for this study (from the 10th January 2020 to the 15th March 2020), so patients in other parts of China, outside Hubei, may have different profiles of demographic, epidemiological, clinical characteristics, laboratory parameters, and image test results. Knowing those profiles may help predict the severity of COVID-19.

Jiangsu, a province in China over 600 km from Hubei without common geographical borders and 80 million population, reported over 600 patients infected with COVID-19. We report here an analysis of nearly all cases in Jiangsu province from the 10th January 2020 to the 15th March 2020 to describe the demographic, epidemiological, clinical, laboratory, and imaging characteristics of cases and to identify risk factors for severe/critically ill COVID-19 presentation. Previous studies were mostly small and descriptive in nature. Our study will use data from the whole Jiangsu province and make inferential statistical analyses by means of multivariate regression model to control for possible confounding factors and identify independent risk factors of becoming a severe/critically ill case.

## Methods

### Study design and population

This is a multicentre retrospective cohort study. All patients were included if they (1) were clinically diagnosed and then confirmed to have COVID-19 in Jiangsu province from the 10th January 2020 up to the 15th March 2020, and (2) fulfilled the diagnostic criteria for the “Diagnosis and Treatment Protocol for Novel Coronavirus Pneumonia (Trial Version 7)” released by National Health Commission & National Administration of Traditional Chinese Medicine of China [23]. The diagnosis of COVID-19 was based on epidemiological history, clinical manifestations, imaging manifestations of pneumonia in CT scans, and laboratory confirmation [23, 24]. Patients without medical records were excluded.

The Ethics Committee of Zhongda Hospital, Affiliated to Southeast University, approved the study protocols (2020ZDSYLL013–P01 and 2020ZDSYLL019–P01). Patient informed consent was waived due to the retrospective study design.

### Data measures

The primary outcome was severe or critically ill within the follow up period. Patients were categorised by disease severity into (1) asymptomatic or mild, or moderate, and (2) severe or critically ill, according to “Diagnosis and Treatment Protocol for Novel Coronavirus Pneumonia (Trial Version 7)” [23]. Asymptomatic infections were defined as the absence of clinical symptoms with a positive nucleic acid test (real-time reverse transcriptase–polymerase chain reaction assay, RT-PCR, for SARS-CoV-2). Mild COVID-19 disease was defined as the presence of mild clinical symptoms without respiratory distress and the absence of imaging manifestations of pneumonia. Moderate disease was defined as the presence of fever, with respiratory symptoms and an image of pneumonia in CT scans. Severe disease was defined as the presence of at least one of the three conditions: respiratory distress, a respiratory rate ≥ 30 beats / min; oxygen saturation in resting state ≤93%; or an arterial blood oxygen partial pressure / oxygen concentration ≤ 300 mmHg (1 mmHg = 0.133 kPa). Critically ill was defined as having respiratory failure requiring mechanical ventilation, shock or combined organ failure requiring intensive care unit (ICU) monitoring and treatment. A clustering onset was defined as the occurrence of two or more confirmed COVID-19 cases in the same cluster/group within 14 days, such as family, community, hospital, working place or public place, etc. A clustering onset could occur due to interpersonal transmission via close contact with or joint exposure to a confirmed COVID-19 case. Others cases not meeting the conditions of the clustering onset were classified as a single onset. All patients were followed up to the 15th March 2020.

Data were collected using case record forms and electronic medical record systems and included demographic, epidemiological, clinical, laboratory, and imaging information provided by the Data Center of Jiangsu Provincial Health Commission without any patient’s personal information. The day of admission to a COVID-19-designated hospital was considered the first day of hospitalisation. The severity of illness was assessed by two physicians. Severity was assessed at days 1, 2, 3, 4, 5, 6, 7 and 14 after admission and patients were followed for up to discharge. ICU admissions were recorded. Imaging grading was performed by two independent radiologists with more than 5 years’ experience in pulmonary imaging. Chest CT axial sections were divided into quadrants (left and right, anterior and posterior) by drawing horizontal and vertical lines through the centre of the chest. Quadrant scores were estimated as the sum of quadrants with pulmonary opacities extending from the proximal to the distal end of the chest and ranged from 0 to 4. Pulmonary opacity was visually estimated and assigned a percentage of pulmonary opacity area in the area of bilateral lungs, rounded to the nearest 5%.

### Statistical analysis

#### Variables

Primary outcome variable was the occurrence of severe/critically ill case. Predictive variables included sex, age, exposure type, types of disease onset, initial symptoms, medical history, vital signs (including body temperature, heart rate (HR), respiratory rate, and peripheral capillary oxygen saturation (SpO_2_)), CT image parameters (including quadrant score and pulmonary opacity), and laboratory parameters (including white blood cells count, neutrophil, lymphocyte, platelet, albumin, creatinine, C-reactive protein, activated partial thromboplastin time, fibrinogen, and D-dimer). Those variables were measured at hospital admission. The other variables including supportive treatments and medical drugs were collected during the follow-up period.

#### Statistical analysis

Continuous variables were described using means (standard deviations, SD) or medians (with inter-quartile range, IQR) by disease severity and were compared using ANOVA or Kruskal-Wallis tests as appropriate. Categorical variables were summarised using frequencies and percentages and compared using Fisher exact tests.

Logistic regression models were used to identify the risk factors for having a severe or critically ill status. Analysis was performed in 2 steps. Firstly, a univariate logistic regression model was fitted for each variable based on completed cases. As there are many potential predictors, we chose the variables for univariate regression analysis if a variable is significant at 5%. Respiratory rate and SpO_2_ were not included in the regression analyses since they were part of criterion for classifying the disease severity. All variables selected for univariate regression analysis were also included in the second stage of the multivariate logistic regression. Missing covariates at admission were imputed with multiple imputation using a Markov Chain Monte Carlo simulation method with 10 iterations. In the logistic regression analysis, odds ratios (ORs) for having a severe or critically ill status for each variable were calculated along with 95% confidence intervals (CIs). A sensitivity analysis was performed on the completed cases. The 2-tailed *P* < 0.05 was considered as statistically significant for all analyses. The analyses were performed using SAS 9.4 (SAS Institute).

## Results

**Fig. 1:**
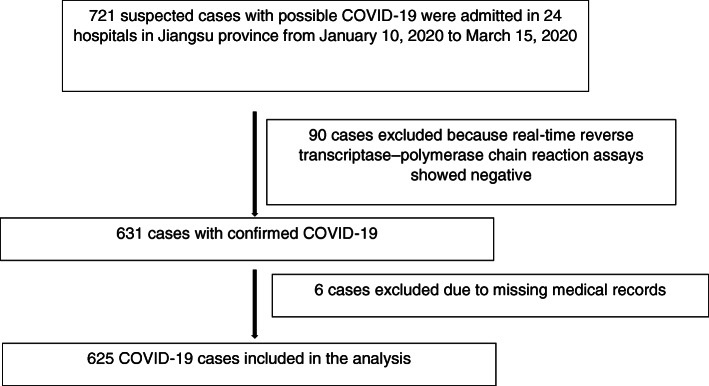
Study flow diagram

From the 10th January 2020 to 15th March 2020, 721 suspected cases with possible COVID-19 were admitted in 24 hospitals in Jiangsu province, China, while 90 cases were excluded because of negative reverse transcriptase–polymerase chain reaction assay. Six hundred thirty-one cases were diagnosed with COVID-19 totally. Of these, 625 (99.0%) had retrievable medical records and were included in the analysis (Fig. 1).

Table 1 describes the characteristics of patients at the time of admission by disease severity and Table S1 provides a more detailed description of the five severity categories. 561 (89.8%) patients were asymptomatic/mild/moderate and 64 (10.2%) patients were severe or critically ill. Patients with severe/critically ill COVID-19 were more likely to be older and to be single onset (i.e. not belong to a cluster of cases in a family/community, etc.). Patients with severe/critically ill presentation were more likely to have a medical history of hypertension and diabetes. Patients with severe/critically ill COVID-19 on admission had higher temperature, faster respiratory rates, lower SpO_2_, and higher CT image quadrant scores and pulmonary opacity percentage (Table 1 and Table S1).

**Table 1:** Demographic and clinical characteristics of patients with COVID-19 at admission^a^

		Severe/Critically ill			
Category	Characteristics	Yes (*N* = 64)	No (*N* = 561)	All (*N* = 625)	*P*-value
Demographic, n/N (%), N, mean (SD)					
	Male	41/64(64.1%)	288/561(51.3%)	329/625(52.6%)	0.0534
	Female	23/64(35.9%)	273/561(48.7%)	296/625(47.4%)	
	Age (year)	64,59.53(13.43)	561,42.72(16.73)	625,44.44(17.19)	<.0001
	≤18 years	0/64(0.0%)	37/561(6.6%)	37/625(5.9%)	<.0001
	19–44 years	6/64(9.4%)	255/561(45.5%)	261/625(41.8%)	
	45–64 years	32/64(50.0%)	216/561(38.5%)	248/625(39.7%)	
	65+ years	26/64(40.6%)	53/561(9.4%)	79/625(12.6%)	
Exposure type, n/N (%)					
	Imported cases	25/64(39.1%)	194/561(34.6%)	219/625(35.0%)	0.4765
	Local cases	39/64(60.9%)	367/561(65.4%)	406/625(65.0%)	
Types of disease onset, n/N (%)					
	Single onset	40/64(62.5%)	270/561(48.1%)	310/625(49.6%)	0.0294
	Clustering onset	24/64(37.5%)	291/561(51.9%)	315/625(50.4%)	
Initial symptoms, n/N (%)					
	Fever	52/64(81.3%)	360/561(64.2%)	412/625(65.9%)	0.0063
	Cough	44/64(68.8%)	300/561(53.5%)	344/625(55.0%)	0.0200
	Sputum	25/64(39.1%)	141/561(25.1%)	166/625(26.6%)	0.0168
Medical history, n/N (%)					
	Hypertension	19/64(29.7%)	72/561(12.8%)	91/625(14.6%)	0.0003
	Diabetes	10/64(15.6%)	30/561(5.3%)	40/625(6.4%)	0.0015
	Stroke	2/64(3.1%)	8/560(1.4%)	10/624(1.6%)	0.2736
Vital signs, N, mean (SD)					
	Temperature (°C)	64,37.30(0.94)	561,37.02(0.70)	625,37.05(0.73)	0.0040
	HR (bpm)	64,89.98(15.06)	561,86.84(13.25)	625,87.17(13.46)	0.0772
	Respiratory rate (breath per min)	64,20.98(4.87)	561,18.87(2.04)	625,19.08(2.56)	<.0001
	SpO_2_ (%)	64,95.53(4.70)	561,97.92(1.15)	625,97.68(1.99)	<.0001
CT image, N, median (IQR)					
	Quadrant score (1–4)	58,4.0(4.0–4.0)	438,2.0(1.0–4.0)	496,2.0(1.0–4.0)	<.0001
	Pulmonary opacity (%)	58,50.0(35.0–70.0)	438,20.0(5.0–30.0)	496,20.0(5.0–40.0)	<.0001
Laboratory test, N, median (IQR)					
	WBC Count (10^9^/L)	52,4.3(3.4–5.8)	461,5.0(4.0–6.3)	513,4.9(3.9–6.2)	0.0440
	Neutrophil (10^9^/L)	52,2.9(2.0–4.4)	455,3.0(2.2–4.0)	507,3.0(2.2–4.0)	0.7435
	Lymphocyte (10^9^/L)	52,0.7(0.5–1.0)	453,1.4(1.0–1.8)	505,1.3(0.9–1.7)	<.0001
	Platelet (10^9^/L)	48,154.0(118.0–185.0)	446,188.5(154.0–222.0)	494,183.5(151.0–219.0)	<.0001
	Albumin (g/L)	48,39.7(34.2–41.4)	432,42.0(38.0–45.7)	480,41.4(38.0–45.1)	<.0001
	Creatinine (umol/L)	49,64.0(51.0–83.0)	427,63.8(51.0–79.0)	476,63.9(51.0–79.0)	0.6950
	C-reactive protein (mg/L)	44,40.1(8.6–92.7)	430,10.0(2.6–19.3)	474,10.0(2.7–22.6)	<.0001
	Activated partial thromboplastin time (s)	54,32.6(28.5–36.5)	459,32.2(27.9–37.4)	513,32.2(28.0–37.2)	0.8158
	Fibrinogen (g/L)	53,4.3(3.2–5.9)	443,3.4(2.7–4.1)	496,3.5(2.7–4.2)	<.0001
	D-dimer (mg/L)	51,0.3(0.2–1.0)	424,0.2(0.1–0.4)	475,0.2(0.1–0.4)	0.0003

^a^Continuous variables: ANOVA or Kruskal-Wallis tests as appropriate; categorical variables: Fisher exact tests

Cases with severe/critically ill presentation were more likely to have increased C-reactive protein, fibrinogen, and D-dimer than asymptomatic/mild/moderate cases. Similarly, severe/critically ill cases had lower white blood cells, lymphocyte, and platelet counts and albumin (Table 1). As expected, severe cases were more likely to use supportive treatments and medical drugs, including antibiotics and antivirals, except interferon (Table 2).

**Table 2:** Clinical management and outcome

		n (%) or median (IQR)			*P*-value
		Severe/Critically ill			
Category	Clinical management and outcome	Yes (*N* = 64)	No (*N* = 561)	All (*N* = 625)	
Supportive treatments	Inotropic and vasoconstrictive agents	5(7.8%)	0(0.0%)	5(0.8%)	<.0001
	Nasal cannula	53(82.8%)	168(29.9%)	221(35.4%)	<.0001
	Mask	12(18.8%)	2(0.4%)	14(2.2%)	<.0001
	High-flow nasal cannula oxygen therapy	24(37.5%)	1(0.2%)	25(4.0%)	<.0001
	Non-invasive ventilation	34(53.1%)	0(0.0%)	34(5.4%)	<.0001
	Intermittent mandatory ventilation	5(7.8%)	0(0.0%)	5(0.8%)	<.0001
	Prone position	17(26.6%)	1(0.2%)	18(2.9%)	<.0001
	Continuous renal replacement therapy	1(1.6%)	0(0.0%)	1(0.2%)	0.1024
	Extracorporeal membrane oxygenation	2(3.1%)	0(0.0%)	2(0.3%)	0.0103
	Lung transplantation	2(3.1%)	0(0.0%)	2(0.3%)	0.0103
Medical drugs	Traditional Chinese medicine	29(45.3%)	69(12.3%)	98(15.7%)	<.0001
	Immunoglobulin	50(78.1%)	106(18.9%)	156(25.0%)	<.0001
	Interferon	47(73.4%)	456(81.3%)	503(80.5%)	0.1363
	Antioxidants	35(54.7%)	117(20.9%)	152(24.3%)	<.0001
	Glucocorticoid	52(81.3%)	90(16.0%)	142(22.7%)	<.0001
	Thymosin	43(67.2%)	101(18.0%)	144(23.0%)	<.0001
	Neurotrophic drugs	21(32.8%)	81(14.4%)	102(16.3%)	0.0005
	Any antibiotics	59(92.2%)	277(49.4%)	336(53.8%)	<.0001
	Any antivirals	64(100%)	516(92.0%)	580(92.8%)	0.0098
Clinical outcome	Death	0(0.0%)	0(0.0%)	0(0.0%)	NC
	Hospital stay	21.5(15.0–29.0)	15.0(12.0–21.0)	16.0(12.0–22.0)	<.0001

None of the patients died and 625 (100%) of 625 patients were discharged by the end of study (15th March 2020). The results from the univariate and multivariate logistic regression analyses are presented in Table 3. Factors independently associated with severe or critically ill infection included age (year) (OR 1.06, 95%CI 1.03–1.09), lymphocyte count (10^9^/L) (OR 0.25, 95%CI 0.08–0.74), and pulmonary opacity in CT (per 5%) on admission (OR 1.31, 95%CI 1.15–1.51). Sensitivity analysis showed that they remained statistically significant in a logistic model with only above three variables based on the completed cases without missing data.

**Table 3:** Factors associated with severe/critically ill in patients with COVID-19: Results from logistic regression analysis

	Univariate analysis^a^		Multivariate analysis^b^	
Variables	Odds ratio (95%CI)	*P*-value	Odds ratio (95%CI)	*P*-value
Age (year)	1.07(1.05,1.09)	<.0001	1.06(1.03,1.09)	<.0001
Single onset	1.80(1.05,3.06)	0.0311	0.92(0.43,1.96)	0.8275
Fever	2.42(1.26,4.64)	0.0078	1.50(0.64,3.54)	0.3542
Cough	1.91(1.10,3.33)	0.0216	1.24(0.54,2.87)	0.6110
Sputum	1.91(1.12,3.27)	0.0183	1.12(0.48,2.60)	0.7994
Hypertension	2.87(1.59,5.18)	0.0005	1.06(0.47,2.40)	0.8874
Diabetes	3.28(1.52,7.07)	0.0025	1.64(0.52,5.22)	0.4004
Temperature (°C)	1.59(1.15,2.19)	0.0046	0.95(0.61,1.47)	0.8133
Lymphocyte (10^9^/L)	0.03(0.01,0.08)	<.0001	0.25(0.08,0.74)	0.0161
Platelet (10^9^/L)	0.99(0.98,0.99)	0.0003	1.00(0.99,1.00)	0.5147
Albumin (g/L)	0.91(0.87,0.96)	0.0002	0.99(0.92,1.07)	0.8344
C-reactive protein (mg/L)	1.02(1.01,1.02)	<.0001	1.00(0.99,1.01)	0.9789
Fibrinogen (g/L)	1.87(1.50,2.32)	<.0001	1.04(0.72,1.49)	0.8327
D-dimer (mg/L)	1.28(1.06,1.55)	0.0088	1.17(0.83,1.66)	0.3625
Quadrant score (1–4)	2.28(1.71,3.05)	<.0001	0.90(0.56,1.47)	0.6811
Pulmonary opacity (per 5%)	1.38(1.28,1.49)	<.0001	1.31(1.15,1.51)	0.0001

^a^Univariate analysis is based on the complete cases without missing value

^b^Multivariate analysis is based on imputed values for missing data in Lymphocyte, Platelet, Albumin, C-reactive protein, Fibrinogen, D-dimer, Quadrant score and Pulmonary opacity using multiple imputation method

## Discussion

In this large multicentre cohort, 64 (10.2%) of 625 patients were severe or critically ill. This proportion of severe or critically ill cases is lower than the 17.7% reported from Wuhan but similar to the 10.4% in Hubei province and higher than the 7.0% reported for areas outside Hubei province [3]. Specifically, it is lower than the figures reported from several case series from Wuhan including 13 (32%) ICU admission among 41 cases with 6 (15%) deaths [25]; 11 (11%) deaths among 99 cases [9]; and 36 (26.1%) ICU admissions among 138 patients, with 6 deaths (4.3%) [10]. The lower proportion than that in Hubei is likely due to several factors, including more adequate medical resources, better disease recognition and testing capacity, earlier identification of asymptomatic and mild cases and a more informed supportive care in COVID-19-designated hospitals. The proportion of critically ill cases in early stage of COVID-19 outbreak in New York City, USA was much higher (22% [257]) [26] than in China, with 14.2% treated in the ICU [27] and 23.6% required mechanical ventilation reported in other studies [28]. Singapore also reported a higher proportion of severe cases in an early study (33.3% [6]) [29]. A more recent study from USA reported 5.8% (7162) of cases suffered from severe COVID-19 [30] which is lower than in China. The proportions of severe COVID-19 in different countries vary a lot, which may result from quite different study designs, the COVID-19 outbreak stages when data were collected, population characteristics, health resources, government response measurements, et al.

Despite this being a hospital-based study, some patients had no symptoms. This is likely due to testing of contacts after the identification of an index case and the policy of hospitalization of all infected individuals at the initial stages of the epidemic, independently of the presence of symptoms. This study found that fever, cough, and sputum were very common among patients with COVID-19 and more frequent in patients with severe or critically ill COVID-19. Similar to COVID-19, fever and cough are the most common symptoms of the other two diseases caused by coronavirus, i.e. severe acute respiratory syndrome (SARS) and Middle East respiratory syndrome (MERS) [31, 32]. Fever is a primary symptom for cytokine storms, with the production of high concentrations of cytokines stimulating abnormally excessive immune responses and inflammation [33–35]. Vital signs showed severe or critically ill patients had higher body temperature and respiratory rate, and lower SpO_2_ on admission. SpO_2_ < 90% has been used as a marker for the use of glucocorticoids during the outbreak [36], and the oxygenation saturation index is associated with ARDS severity and increased mortality [37, 38].

Gender had no effect on severity of COVID-19 patients. Although early reports from Wuhan indicated more men than women had severe COVID-19, recent studies reported similar proportions of men and women admitted to ICUs [9, 11, 25], suggesting gender differences disappeared with higher incidence. Earlier reports may have included more males due to a higher occupational infection risk for males in the markets and congregation places [10].

Our study found that age was independently associated with severe or critically ill presentation. Age is a well-established factor for severe/critically ill COVID-19 for individuals > 60, and especially over 80 years old [39, 40]. Similarly, previous reports have indicated patients in ICUs are older than non-ICU patients [10], and that CFRs are higher among older individuals [9, 11, 25]. Older patients also have faster disease progression than younger patients [41], which is similar to the MERS and SARS presentations, in which, older age (> 60 or 45) is associated with disease severity (MERS) [42, 43], and mortality [44, 45]. Older age reflects a greater likelihood of underlying medical conditions such as hypertension and diabetes, which predisposes to immunological vulnerabilities. Also, age-related immunosenescence may also contribute to the severe disease [46].

In Wuhan, many asymptomatic patients had abnormal lung CT findings on admission, which then progressed to diffuse ground-glass opacities and consolidation [47]. In Jiangsu, several asymptomatic cases also had radiological changes presented as low quadrant scores and pulmonary opacity scores on admission and severe/critically-ill cases had higher CT quadrant and pulmonary opacity scores than moderate cases. Our study also identified pulmonary opacity as an independent predictor of severe/critical illness. This is consistent with the previous study reporting that the CT visual quantitative evaluation of acute lung inflammatory lesions involving each lobe in severe or critical cases was significantly higher than less severe cases [48].

We found severe or critically ill patients had more obvious damage of white blood cells and immune cells such as lymphocytes with lymphocytes identified as an independent predictor of more severe disease. COVID-19 may cause the reduced T lymphocytes, especially CD4 + T and CD8 + T cells, leading to reduced IFN-γ production, which may be related to the severity of disease [49]. In addition, severe or critically ill patients showed more serious organ dysfunction like reduced albumin on admission which may be a sign of reduced liver production and increased gastrointestinal or renal loss, and increased fibrinogen on admission responding to systemic inflammation and tissue damage, and more fierce inflammatory response presented as much higher level of inflammatory markers, such as C-reactive protein [50–52].

This study has several strengths. Firstly, this is one of the largest studies describing the clinical characteristics of patients with COVID-19 and risk factors for severe/critically ill infection outside the Wuhan, epicentre of the epidemic in China at the time of data collection for this study (from the 10th January 2020 to the 15th March 2020). Secondly, the cohort includes almost all COVID-19 cases in the province, which may have reduced selection bias. Thirdly, Jiangsu province, which is far from Hubei, provides an opportunity to assess the demographic, epidemiological, clinical, laboratory, and imaging features of cases imported from other provinces and local cases. Fourthly, asymptomatic and mild cases were included, which provides a more comprehensive description of the characteristics of COVID-19 cases with a broad spectrum of disease severity.

There are also limitations that need mentioning. Firstly, laboratory and radiological data had a large amount of missing data preventing their integration in the analysis. Secondly, the predictive factors identified may be subject to uncontrolled confounders by unknown/unmeasured factors such as occupation and pregnancy. Medical staff and pregnant women may have different severity profiles. Thirdly, this is a retrospective observational study and data may be susceptible to measurement and information bias.

## Conclusion

In conclusion, this study demonstrates that patients with COVID-19 in Jiangsu had a low rate of severe or critically ill presentation, with no deaths recorded. The COVID-19 severity is associated with epidemiological and clinical characteristics, laboratory test, and radiological findings. Age, lymphocyte count, and pulmonary opacity in CT on admission were independently associated with risk of severe or critically ill COVID-19 presentation.

## Supplementary information

**Additional file 1: Table S1.** Demographic and clinical characteristics of patients at admission by disease severity*.

## Data Availability

The datasets used and analysed during the current study are available from the corresponding author on reasonable request.

## Abbreviations

COVID-19: Coronavirus Disease-2019
SARS-CoV-2: Severe Acute Respiratory Syndrome Coronavirus-2
SpO_2_: Peripheral capillary oxygen saturation
CT: Computer tomography
WHO: World Health Organization
CFR: Case fatality rate
EU: The European Union
EEA: European Economic Area
ARDS: Acute respiratory distress syndrome
RT-PCR: Reverse transcriptase–polymerase chain reaction assay
ICU: Intensive care unit
HR: Heart rate
SD: Standard deviation
IQR: Inter-quartile range
OR: Odds ratio
CI: Confidence interval
SARS: Severe acute respiratory syndrome
MERS: Middle East respiratory syndrome
