# Supplementary material for: Clinical characteristics and risk factors of patients with severe COVID-19 in Jiangsu province, China: a retrospective multicentre cohort study
Source: BMC Infect Dis. 2020 Aug 6;20:584. doi: 10.1186/s12879-020-05314-x (PMC7407434; doi:10.1186/s12879-020-05314-x)
Supplement: Supplementary file 1 — Additional file 1: Table S1. Demographic and clinical characteristics of patients at admission by disease severity*. [file 12879_2020_5314_MOESM1_ESM.docx]

Table S1: Demographic and clinical characteristics of patients at admission by disease severity*

|  | | Disease severity, total, mean(SD) or total, median(IQR) or n/total(%) | | | | | |  |
| --- | --- | --- | --- | --- | --- | --- | --- | --- |
| Category | Characteristics | Asymptomatic (N=24) | Mild (N=35) | Moderate (N=502) | Severe (N=30) | Critically ill (N=34) | All (N=625) | P-value |
| Demographic | Male | 11/24(45.8%) | 13/35(37.1%) | 264/502(52.6%) | 16/30(53.3%) | 25/34(73.5%) | 329/625(52.6%) | 0.0416 |
|  | Age (year) | 24,36.75(24.91) | 35,27.42(16.37) | 502,44.07(15.70) | 30,57.73(14.50) | 34,61.12(12.40) | 625,44.44(17.19) | <.0001 |
| Exposure type | Imported cases | 3/24(12.5%) | 11/35(31.4%) | 180/502(35.9%) | 14/30(46.7%) | 11/34(32.4%) | 219/625(35.0%) | 0.0936 |
|  | Local cases | 21/24(87.5%) | 24/35(68.6%) | 322/502(64.1%) | 16/30(53.3%) | 23/34(67.6%) | 406/625(65.0%) |  |
| Types of disease onset | Single onset | 7/24(29.2%) | 4/35(11.4%) | 259/502(51.6%) | 19/30(63.3%) | 21/34(61.8%) | 310/625(49.6%) | <.0001 |
|  | Clustering onset | 17/24(70.8%) | 31/35(88.6%) | 243/502(48.4%) | 11/30(36.7%) | 13/34(38.2%) | 315/625(50.4%) |  |
| Initial symptoms | Fever | 0/24(0.0%) | 11/35(31.4%) | 349/502(69.5%) | 27/30(90.0%) | 25/34(73.5%) | 412/625(65.9%) | <.0001 |
|  | Cough | 0/24(0.0%) | 18/35(51.4%) | 282/502(56.2%) | 19/30(63.3%) | 25/34(73.5%) | 344/625(55.0%) | <.0001 |
|  | Sputum | 0/24(0.0%) | 5/35(14.3%) | 136/502(27.1%) | 12/30(40.0%) | 13/34(38.2%) | 166/625(26.6%) | 0.0005 |
| Medical history | Hypertension | 3/24(12.5%) | 1/35(2.9%) | 68/502(13.5%) | 7/30(23.3%) | 12/34(35.3%) | 91/625(14.6%) | 0.0018 |
|  | Diabetes | 3/24(12.5%) | 0/35(0.0%) | 27/502(5.4%) | 4/30(13.3%) | 6/34(17.6%) | 40/625(6.4%) | 0.0056 |
| Vital signs | Temperature (°C) | 24,36.63(0.40) | 35,36.79(0.62) | 502,37.06(0.71) | 30,37.09(0.80) | 34,37.49(1.02) | 625,37.05(0.73) | <.0001 |
|  | HR (bpm) | 24,85.33(14.87) | 35,89.54(15.80) | 502,86.73(12.98) | 30,86.50(14.01) | 34,93.06(15.49) | 625,87.17(13.46) | 0.0699 |
|  | MAP (mmHg) | 24,92.08(12.32) | 35,92.31(12.40) | 501,97.38(10.33) | 30,93.78(11.48) | 34,98.40(10.00) | 624,96.77(10.67) | 0.0035 |
|  | Respiratory rate (breath per min) | 24,19.0(18.0-20.0) | 35,18.0(18.0-20.0) | 502,18.0(18.0-20.0) | 30,19.0(18.0-22.0) | 34,20.0(18.0-23.0) | 625,19.0(18.0-20.0) | 0.0081 |
|  | SpO_2_ (%) | 24,98.0(98.0-99.0) | 35,98.0(98.0-98.0) | 502,98.0(98.0-99.0) | 30,98.0(95.0-98.0) | 34,97.0(93.0-98.0) | 625,98.0(97.0-99.0) | <.0001 |
| CT image | Quadrant score (1-4) | 14,0.0(0.0-1.0) | 22,0.0(0.0-0.0) | 402,2.0(1.0-4.0) | 29,4.0(3.0-4.0) | 29,4.0(4.0-4.0) | 496,2.0(1.0-4.0) | <.0001 |
|  | Pulmonary opacity (%) | 14,0.0(0.0-10.0) | 22,0.0(0.0-0.0) | 402,20.0(5.0-35.0) | 29,50.0(30.0-70.0) | 29,60.0(50.0-80.0) | 496,20.0(5.0-40.0) | <.0001 |

*Continuous variables: ANOVA or Kruskal-Wallis tests as appropriate; categorical variables: Fisher exact tests.
